# Chikungunya Virus and Its Envelope Protein E2 Induce Hyperalgesia in Mice: Inhibition by Anti-E2 Monoclonal Antibodies and by Targeting TRPV1

**DOI:** 10.3390/cells12040556

**Published:** 2023-02-09

**Authors:** Carina Z. Segato-Vendrameto, Camila Zanluca, Amanda Z. Zucoloto, Tiago H. Zaninelli, Mariana M. Bertozzi, Telma Saraiva-Santos, Camila R. Ferraz, Larissa Staurengo-Ferrari, Stephanie Badaro-Garcia, Marília F. Manchope, Amanda M. Dionisio, Felipe A. Pinho-Ribeiro, Sergio M. Borghi, Ana Luiza Pamplona Mosimann, Rubia Casagrande, Juliano Bordignon, Victor Fattori, Claudia N. Duarte dos Santos, Waldiceu A. Verri

**Affiliations:** 1 Laboratory of Pain, Inflammation, Neuropathy, and Cancer, Department of Pathology, Londrina State University, Londrina 86057-970, PR, Brazil; 2 Laboratory of Molecular Virology, Carlos Chagas Institute/Fiocruz PR, Curitiba 81310-020, PR, Brazil; 3 Division of Dermatology, Department of Medicine, Washington University School of Medicine, St. Louis, MO 63110, USA; 4 Center for Research in Health Sciences, University of Northern Paraná, Londrina 86041-140, PR, Brazil; 5 Department of Pharmaceutical Sciences, University Hospital (Health Science Centre), Londrina State University, Londrina 86057-970, PR, Brazil; 6 Vascular Biology Program, Boston Children’s Hospital, Harvard Medical School, 1 Blackfan Circle, Karp Research Building 11.006C, Boston, MA 02115, USA

**Keywords:** Chikungunya virus, E2 envelope protein, monoclonal antibody, joint pain, TRPV1

## Abstract

Chikungunya virus is an arthropod-borne infectious agent that causes Chikungunya fever disease. About 90% of the infected patients experience intense polyarthralgia, affecting mainly the extremities but also the large joints such as the knees. Chronic disease symptoms persist for months, even after clearance of the virus from the blood. Envelope proteins stimulate the immune response against the Chikungunya virus, becoming an important therapeutic target. We inactivated the Chikungunya virus (iCHIKV) and produced recombinant E2 (rE2) protein and three different types of anti-rE2 monoclonal antibodies. Using these tools, we observed that iCHIKV and rE2 protein induced mechanical hyperalgesia (electronic aesthesiometer test) and thermal hyperalgesia (Hargreaves test) in mice. These behavioral results were accompanied by the activation of dorsal root ganglia (DRG) neurons in mice, as observed by calcium influx. Treatment with three different types of anti-rE2 monoclonal antibodies and absence or blockade (AMG-9810 treatment) of transient receptor potential vanilloid 1 (TRPV1) channel diminished mechanical and thermal hyperalgesia in mice. iCHIKV and rE2 activated TRPV1+ mouse DRG neurons in vitro, demonstrating their ability to activate nociceptor sensory neurons directly. Therefore, our mouse data demonstrate that targeting E2 CHIKV protein with monoclonal antibodies and inhibiting TRPV1 channels are reasonable strategies to control CHIKV pain.

## 1. Introduction

Chikungunya virus (CHIKV) is an arthritogenic alphavirus, an arthropod-borne infectious agent responsible for causing Chikungunya fever disease [1,2]. First isolated in Tanzania in 1952 [3], countries afflicted by CHIKV attracted worldwide attention when it resurged, causing a massive outbreak since 2006, affecting the Reunion Island and, more recently, affecting the Caribbean, South America, and Europe [4,5,6].

Two strains of *Aedes* mosquitoes are capable of beginning the cycle of transmission, *A. aegypti* and *A. albopictus* [1,7]. After the bite of CHIKV-infected female mosquitoes, there is a silent incubation period lasting about 2–4 days (range 1–12 days), after which not all individuals infected with the virus will develop symptoms [8]. Those who develop the symptoms can experience it first in the acute stage, which is characterized as an abrupt clinical onset of crippling joint pain, high fever, and rash, being easily confused with dengue [8,9,10,11]. CHIKV patients may also present characteristic impaired ambulation [12]. About 90% of the infected patients experience intense myalgia and polyarthralgia, mainly affecting the extremities (ankles, wrists, phalanges) but also the large joints of the knees and shoulders, and usually, both the left and right joints are affected [3,8,13]. In some cases, chronic disease may be established, with symptoms persisting even after viremia is undetectable [9,14,15,16,17,18].

The Chikungunya virus genome consists of a single-strand RNA molecule encoding four nonstructural proteins (nsP1-4) that are required for viral replication and five structural proteins (C, E1, E2, E3, and a 6K protein) [1], E1 being a class II viral fusion protein, while E2 mediates receptor attachment [19]. The use of structural proteins as targets for immunization and therapy against CHIKV infection has received attention over the last few years [7,20,21,22,23]. Recent results have been achieved by researchers using recombinant E2 protein to develop different candidate vaccines against CHIKV. A recent study showed that immunization of AG129 mice using a recombinant Modified Vaccinia Ankara (MVA) expressing E2 or E3E26KE1 elicited neutralizing antibodies in all animals and provided 100% protection against lethal disease. They concluded that MVA expressing the E2 glycoprotein of CHIKV represents an immunogenic and effective candidate vaccine against CHIKV infections [24].

The envelope and capsid proteins of CHIKV are involved in the capacity of the virus to elicit both cellular and humoral immune responses once the E2 protein epitope E2EP3 is identified by activated CD4^+^ T cells inducing IFN-γ production and culminating in joint swelling and inflammation, which was prevented by treatment with the Fingolimod, an agonist of sphingosine 1-phosphate receptor [14,25]. These results show the important participation of E2 protein in joint inflammation induced by CHIKV. On the other hand, concerning humoral immune response, it was verified that E1/E2 and capsid proteins are the main antigens driving the response in both patients with acute and chronic disease [14]. Thus, targeting the E2 protein or the effect elicited by this protein could represent a strategy to treat symptoms such as inflammation and avoid collateral effects such as swelling and pain.

Regarding current treatment, there is no effective antiviral specific to Chikungunya. Treatment is purely symptomatic and based on non-salicylate analgesics and non-steroidal anti-inflammatory drugs [13]. Patients usually receive fluids and medicines to relieve symptoms of fever and aching, such as ibuprofen, naproxen, acetaminophen, or paracetamol. Studies have reported efficacy in the use of antivirals such as interferon-α (in vitro) and ribavirin (a clinical trial with 10 patients per group) on chikungunya virus infection and, despite the use of chloroquine being suggested, trials have failed to confirm its efficacy on CHIKV associated arthralgia [1,13].

On the other hand, monoclonal antibodies (mAbs) against E2 protein have been studied as potential therapies. Two mAbs specific for the CHIKV envelope protein E2 neutralize CHIKV infection in vitro, showing potential to be therapeutic tools against CHIKV infection [26]. A human neutralizing mAb targeting the acid-sensitive region in CHIKV E2, when administered therapeutically at 8 and 18 h after infection, completely protected 100% of mice, confirming its potential for prophylactic and therapeutic protection against viremia also in vivo [19].

Recently, dominant CD4^+^ T cell epitopes were identified in nsP1 and E2 viral antigens capable of recapitulating joint swelling, vascular leakages, edema, inflammation, and necrosis of the muscles when primary CD4^+^ T cell pathogenic epitope-specific cell lines were transferred into virus-infected T cell receptor-deficient (TCR−/−) mice [25]. Thus, the E2 protein is involved in the host’s inflammatory response against the virus. Considering that pain seems to be one of the most critical symptoms in CHIKV disease and due to the importance of the structural proteins as a potential therapeutic and prophylactic target, we aimed to investigate the participation of the E2 protein-induced hyperalgesia, as well as the possible mechanisms involved, such as targeting TRPV1. We also investigated the ability of monoclonal antibodies treatment to control inactivated CHIKV (iCHIKV) or recombinant E2 (rE2)-induced pain to validate the significance of E2 protein as a target in CHIKV pain.

## 2. Materials and Methods

### 2.1. Animals

We used male Swiss (25–30 g) obtained from the Londrina State University (Paraná, Brazil), and C57BL/6 and TRPV1−/− mice (20–25 g) from Ribeirão Preto Medical School, São Paulo, Brazil, to perform the experiments. The animals were housed at 22 ± 2 °C under a 12-h light/12-h dark cycle with free access to food and water and acclimatized to the laboratory for at least 1 h before testing. All performed experiments were approved by the Ethics Committee on Animal Research of the Londrina State University (process number 13216.2017.97 of 12/07/2017). Experiments were carried out in accordance with the current guidelines for the care of laboratory animals and the ethical guidelines for investigations of experimental pain in conscious animals [27] and the International Association for the Study of Pain (IASP).

### 2.2. Inactivation of Chikungunya Virus

CHIKV BR_2015/15010 was isolated from the serum of a patient with chikungunya disease from Northeast Brazil in 2015. The virus was amplified, titrated by foci-forming assay in C6/36 cells [28], and inactivated using β-propiolactone (0.025%, 72 h, 4 °C). The inactivated virus was concentrated by PEG 7%/NaCl 2.3% precipitation and purified using a sucrose cushion. A noninfected control (Mock) was prepared in the same manner from β-propiolactone inactivated C6/36 cell-culture supernatant.

### 2.3. Cloning, Expression, and Purification of CHIKV rE2 Protein

**Figure 1:**
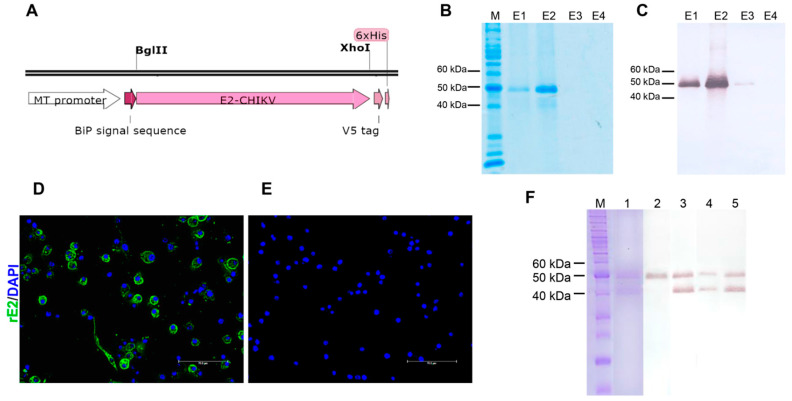
Expression and purification of Chikungunya virus (CHIKV) recombinant E2 (rE2) protein and its identification by monoclonal antibodies. (**A**) Schematic construction design of the rE2 gene in pMT/Bip/V5-His A vector. The rE2 expression and characterization were confirmed by SDS-PAGE (**B**) and Western blot using anti-V5 antibodies (**C**). Proteins were stained with an anti-V5 antibody, followed by anti-mouse IgG conjugated to alkaline phosphatase. M: molecular weight marker; E1–E4: elution fractions 1 to 4. The three monoclonal antibodies recognized the rE2 (green) protein by indirect immunofluorescence (**D**) and Western blot (**F**). (**E**) represents the negative control of immunofluorescence analysis. (**D**) anti-rE2 protein mAb; the three mAbs exhibited the same staining pattern (representative image is shown). (**E**) a non-related anti-hantavirus mAb (clone 572/7A) was used as an unrelated antibody-negative control. For Western blot analysis, rE2 was subjected to 13% SDS-PAGE and electroblotted onto nitrocellulose membranes. Proteins were stained with the mAbs (**F**—lines 3–5), followed by anti-mouse IgG conjugated to alkaline phosphatase. The anti-V5 antibody was used as a positive control (**F**—line 2). M: molecular weight marker; line 1: SDS-PAGE of the proteins subjected to Western blot.

The recombinant ectodomain of CHIKV E2 protein was expressed in *Drosophila melanogaster* S2 cells because this system allows posttranslational modifications, such as glycosylation. The ectodomain of the CHIKV E2 protein gene was determined based on the results by Cho and colleagues [29] and synthesized by GenScript (Piscataway, NJ, USA) with codon optimization for expression in *Drosophila melanogaster* cells. The synthetic gene was inserted into pMT/Bip/V5-His A (Invitrogen) via the Bgl II, and Xho I restriction sites (Figure 1A). The recombinant expression vector was sequenced at Macrogen Inc. services (Seoul, Republic of Korea) for in-frame insertion verification. S2 cells (Invitrogen) were co-transfected with the recombinant expression vector, and pCoBlast (at a ratio of 19:1) using CaCl_2_ and selected with 25 µg/mL of blasticidin S in Schneider’s Drosophila Medium (Gibco/Invitrogen) supplemented with 10% fetal bovine serum and 100 IU/μg/mL penicillin/streptomycin (Gibco/Invitrogen) at 28 °C. The stably transfected cells were cultured in SF-900 II medium (Invitrogen) with 100 IU/μg/mL penicillin/streptomycin. Cells were seeded at a density of 1 × 10^6^ cells/mL, and the rE2 protein expression was induced by 700 µM of CuSO_4_ on day 2 of culture for 24 h. The rE2 protein was purified from the cell-culture supernatant by immobilized metal affinity chromatography. Briefly, the supernatant containing the rE2 protein was dialyzed against ligation buffer (50 mM NaH_2_PO_4_.H_2_O, 500 mM NaCl; pH 8.0), followed by incubation with Ni-NTA agarose resin (Qiagen), and packaging in a Poly-Prep column (BioRad, Hercules, CA, USA). Then, after washing with 5 column volumes of buffer containing 20 mM imidazole, the rE2 protein was eluted with 500 mM imidazole in buffer containing 50 mM NaH_2_PO_4_.H_2_O and 300 mM NaCl (pH 8.0). After verification, the most concentrated elution fractions were mixed and stored at −20 °C. The mock control was prepared from the supernatant of S2 cells induced by 700 µM of CuSO_4_ and purified following the same protocol. The quality of rE2 expression was confirmed by SDS-PAGE and Western blot using anti-V5 antibodies. Briefly, proteins were mixed with Laemmli sample buffer, boiled for 5 min, and loaded into 13% SDS-PAGE gels [29]. Proteins were transferred to nitrocellulose membranes (GE-Healthcare, Little Chalfont, UK). Membranes were incubated with 5% non-fat milk in TBS (20 mM Tris, 137 mM NaCl, pH 7.6) containing 0.05% Tween 20, followed by anti-V5 antibodies, and then by anti-mouse IgG conjugated to alkaline phosphatase (Promega, Madison, WI, USA). All incubation steps were conducted for 1 h at room temperature. The reaction was developed using a solution of NBT (nitroblue tetrazolium) and BCIP (5-bromo-4-chloro-3-indolyl-phosphate) (Promega).

### 2.4. Anti-rE2 Antibodies Synthesis

Hybridomas secreting anti-rE2-CHIKV monoclonal antibodies (mAbs) were obtained by the fusion of mouse myeloma cell line P3X63Ag8.653 (ATCC CRL-1580) and splenocytes from Balb/c mice immunized with the rE2-CHIKV protein following the protocol previously described [30]. The production of anti-rE2 antibodies was approved by the Ethics Committee for Animal Research of the Federal University of Paraná, process number 23075.082918/2011-51 of 07/06/2011. Three hybridomas were obtained. MAbs were purified on a protein-G column (GE-Healthcare) and quantified using the Micro BCA Protein Assay kit (Thermo Scientific, Waltham, MA, USA) according to the manufacturer’s instructions. The recognition of rE2 protein by the mAbs was confirmed by Western blot and immunofluorescence. For immunofluorescence, the S2 cells expressing the CHIKV rE2 protein were fixed in methanol:acetone and stained with the mAbs, followed by Alexa-Fluor 488-conjugated anti-mouse immunoglobulin. Nuclei were counter-stained with DAPI.

### 2.5. Experimental Protocols

Mice received intra-articular (i.a.) injection of inactivated Chikungunya virus (iCHIKV; 1, 10, 100 and 1000 FFU/10 µL saline), recombinant E2 (rE2) protein (30 and 100 ng/10 µL saline) or Mock (negative control, 10 µL, please see Section 2.2 and Section 2.3). The mechanical hyperalgesia was measured 1, 3, 5, and 7 h after the stimulus injection and daily for seven days. The best virus and protein doses were chosen for the following experiments based on mechanical hyperalgesia. Aiming to evaluate the possibility of modulating iCHIKV and rE2 protein-induced hyperalgesia, co-treatments with anti-rE2 antibodies (mAb1, mAb2, mAb3, 1 and 5 µg, i.a.) were performed with the inactivated virus or with the protein (mixtures were prepared immediately before co-injection). Mechanical hyperalgesia was evaluated 1, 3, 5, and 7 h after the co-treatment and daily for seven days. To evaluate the participation of the TRPV1 receiver in this model, we injected iCHIKV 100 FFU, rE2 100 ng, or Mock in TRPV1−/− mice and measured the mechanical hyperalgesia 1, 3, 5, and 7 h and the thermal hyperalgesia 7 h after the stimulus injection. We also treated C57BL/6 mice with TRPV1 antagonist AMG-9810 (100 nmol, intrathecal, Cayman Chemical, Ann Arbor, Michigan, USA) to confirm the importance of this receptor in the iCHIKV or rE2-induced pain. Based on TRPV1 mechanical hyperalgesia peak, thermal hyperalgesia was evaluated 7 h after the stimulus. Ipsilateral dorsal root ganglia (DRG from L4-L6 spinal cord segments) were also dissected for calcium imaging using confocal microscopy. This approach was used to investigate whether iCHIKV and rE2 activate capsaicin-responsive neurons, which are TRPV1+ neurons.

### 2.6. Mechanical Hyperalgesia

Mechanical hyperalgesia was tested in mice, as previously reported [31]. Briefly, the test consists of evoking a tibiotarsal flex reflex with a hand-held force transducer (electronic von Frey aesthesiometer; Insight, Ribeirão Preto, SP, Brazil) adapted with a non-nociceptive tip probe with an area size of 4.15 mm^2^. The investigator was trained to apply the tip perpendicularly to the central area of the plantar surface, inducing the flex of the hind limb joints. The results were expressed as the flex-elicited withdrawal threshold (in grams). The intensity of the hyperalgesia was quantified as the change in pressure applied by subtracting the mean of the 3 values obtained at different times after the stimulus injection observed from the mean of the three values before the stimulus injection [32].

### 2.7. Thermal Hyperalgesia

Thermal hyperalgesia responses were determined as described by Hargreaves [33]. Briefly, mice were individually confined to plexiglass chambers, and a high-intensity projector bulb was positioned to deliver beneath the right hind paw to a direct thermal stimulus. The withdrawal latency period of the injected paw was determined with an electronic clock circuit. Animals were evaluated at 0 and 7 h after the stimulus, and a cutoff latency of 30 s was used to prevent tissue damage. Results are expressed as paw-withdrawal latency change (s).

### 2.8. Calcium Imaging

Adult wild-type Swiss mice were injected with iCHIKV, rE2, or Mock and euthanized with isoflurane after 7 h of stimulation. The L4-L6 dorsal root ganglia were dissected into DMEM (Invitrogen, Darmstadt, Germany), dissociated in collagenase A (1 mg/mL)/dispase II (2.4 U/mL) (Roche Applied Sciences 04942078001) in HEPES-buffered saline (Sigma 51558-50ML) (5 mM CaCl_2_) at 37 °C for 20 min. The dorsal root ganglia were then crushed with pipettes of decreasing size, and dissociated neurons were plated and incubated with laminin for 45 min at 37 °C. DRGs were then loaded with 1.2 μM of Fluo-4AM in Neurobasal-A medium, incubated for 30 min at 37 °C, washed with HBSS, and imaged on a Confocal Microscope (TCS SP8, Leica Microsystems, Mannheim, Germany). To assess neuron activation, DRG plates were recorded for 2 min of initial reading to achieve fluorescence values with the LAS X software (Leica Microsystems). The color scale indicates the fluorescence intensity, where blue is low and red is the maximum intensity [34]. 

### 2.9. Statistical Analysis

The results presented are representative of two independent experiments and are expressed as the mean ± SEM (*n* = 6 per group per experiment). Two-way ANOVA followed by Bonferroni’s test was performed to evaluate the differences between responses. Statistical differences were considered as significant at *p* < 0.05.

## 3. Results

### 3.1. Expression and Purification of CHIKV rE2

To obtain soluble and glycosylated recombinant protein, the CHIKV rE2 protein was expressed in the *Drosophila* expression system. S2 cells were transfected with the recombinant expression vector (codon-optimized E2 gene cloned into pMT/Bip/V5-His A) and selected with blasticidin S (Figure 1A). rE2 protein expression was induced with 700 µM of CuSO4, and proteins in the supernatant were purified by Ni-NTA affinity chromatography. SDS-PAGE analysis and Western blot with anti-V5 antibodies exhibited a molecular weight of ~45 kDa (Figure 1B,C). The protein was eluted mainly in the fractions E1 and E2 (Figure 1B,C), which were pooled and stored for further analysis. Three mouse anti-rE2 protein mAbs were produced. The identification of the recombinant protein was confirmed by indirect immunofluorescence (Figure 1D,E). The reactivity with the mAbs showed that most of the transfected cells were expressing the recombinant protein E2 (in green) (Figure 1D) but not in the negative staining control (transfected cells expressing the recombinant protein E2 stained with the non-related anti-hantavirus mAb-clone 572/7A; Figure 1E). In addition, Western blot analysis in denaturing gel conditions indicates that the mAbs recognized a linear rE2 protein epitope (Figure 1F). In addition, the anti-rE2 mAbs revealed that the two bands that appeared in the gel correspond to the rE2 protein. The lowest molecular weight band matches with the rE2 protein lacking its C-terminus since it was recognized by the anti-rE2 mAbs (Figure 1F—lines 3–5) but not by the anti-V5 antibody (Figure 1F—line 2).

### 3.2. iCHIKV and rE2 Protein Induce Mechanical Hyperalgesia in Mice

**Figure 2:**
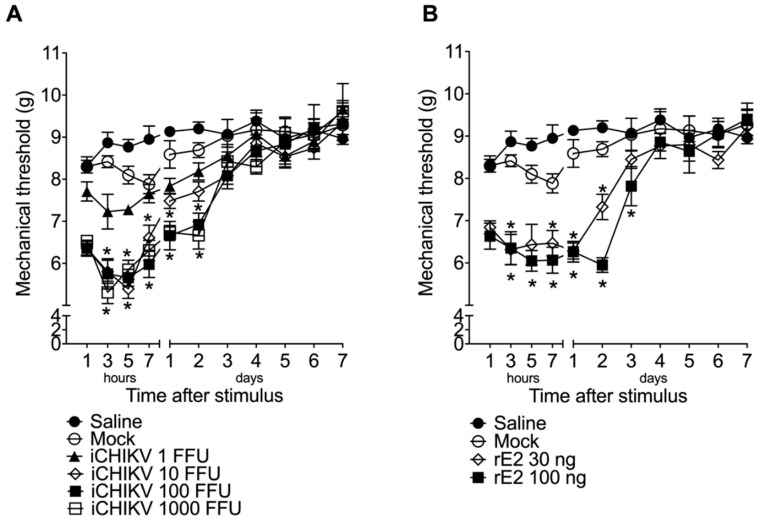
iCHIKV and rE2 protein induce mechanical hyperalgesia in mice. Animals received intra-articular (i.a.) injections of iCHIKV (1, 10, 100, and 1000 FFU, 10 µL), rE2 (30 or 100 ng, 10 µL), or Mock (control, 10 µL). The mechanical hyperalgesia (**A**,**B**) was evaluated 1–7 h after injection and in the following seven days. Results are presented as mean ± SEM of six mice per group per experiment and are representative of two independent experiments. * *p* < 0.05 compared to Mock group; ANOVA followed by Tukey’s test.

Compared to Mock and saline, significant mechanical hyperalgesia was observed starting at the 3rd hour after iCHIKV injection and, with the highest doses, maintained its levels until day two (Figure 2A). There is no significant difference between the doses of 100 and 1000 FFU. Therefore, we chose the lower one to perform the next experiments. As the inactivated virus was capable of inducing significant hyperalgesia, we tested whether the rE2 protein was involved in this process of inducing articular pain. We found that the highest dose of recombinant protein also induced significant hyperalgesia starting at the 3rd hour and maintaining until day three (Figure 2B). We chose the dose of 100 ng to perform the next experiments.

### 3.3. DRG Neurons from iCHIKV and rE2-Stimulated Mice Presented Higher Baseline Levels of Calcium

**Figure 3:**
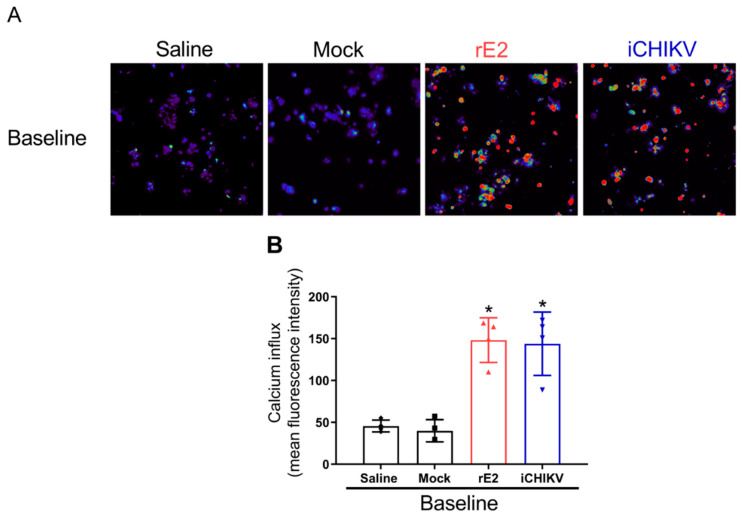
DRG neurons from iCHIKV and rE2-stimulated mice are activated. Seven hours after intra-articular (i.a.) injection of iCHIKV (100 FFU, 10 µL), rE2 (100 ng, 10 µL), saline or Mock (control, 10 µL), DRGs were dissected for calcium imaging using Fluo-4AM. Panel (**A**) displays representative fields of baseline fluorescence of DRG neurons dissected from saline or mock control mice and rE2 or iCHIKV-stimulated mice. Absent calcium levels are shown in blue, and increasing concentrations of calcium are shown by the change in color spectra up to red. Panel (**B**) shows the mean fluorescence intensity of calcium influx on the baseline (0-s mark). Image resolution: 288 × 220 mm (96 × 96 DPI). Results are expressed as mean ± SEM, *n* = 4 DRG plates (each plate is a neuronal culture pooled from six mice) per group per experiment, two independent experiments (* *p* < 0.05 vs. saline and mock; one-way ANOVA followed by Tukey’s post-test).

DRG neurons from iCHIKV and rE2-stimulated mice presented a higher baseline level of calcium influx than DRG neurons from Mock and saline groups (Figure 3A,B). Increases in calcium influx are considered indicative of DRG neuron activation [35]. Therefore, our data suggest that iCHIKV and rE2 induce pain due to peripheral sensitization of nociceptive neurons.

### 3.4. Monoclonal Antibodies Targeting rE2 Reduce rE2 and iCHIKV-Induced Mechanical Hyperalgesia

**Figure 4:**
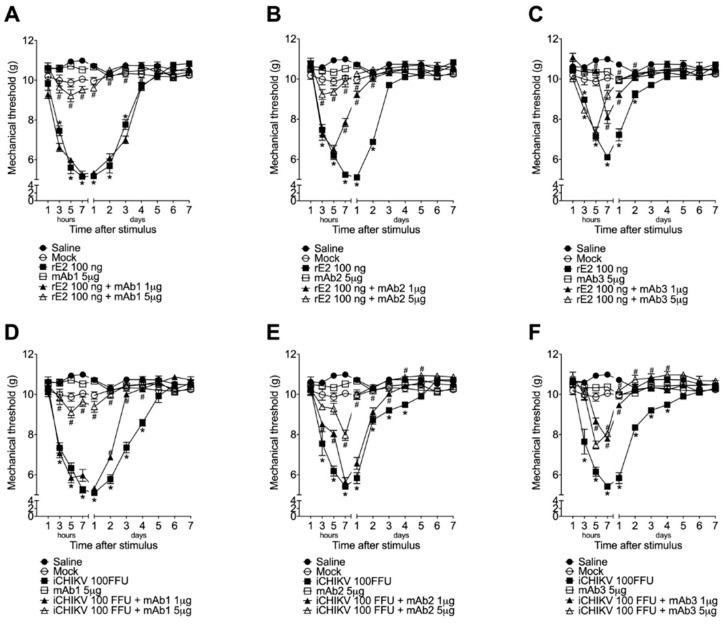
Monoclonal antibodies against E2 protein inhibit iCHIKV and rE2 protein-induced mechanical hyperalgesia. mAb1 (**A**,**D**), mAb2 (**B**,**E**), and mAb3 (**C**,**F**) (1 or 5 µg, 5 µL) were co-injected (intra-articular) with rE2 (100 ng, 5 µL, **A**–**C**), or iCHIKV (100 FFU, 5 µL, **D**–**F**) or Mock (control, 10 µL). The mechanical hyperalgesia was evaluated 1–7 h after injection and daily for seven days. Results are presented as mean ± SEM of six mice per group per experiment and are representative of two independent experiments. * *p* < 0.05 compared to Mock group; # *p* < 0.05 compared to rE2 group. ANOVA followed by Tukey’s test.

The dose of 5 µg of mAb1 and the doses of 1 and 5 µg of mAb2 and mAb3 showed efficacy in inhibiting rE2-induced mechanical hyperalgesia significantly (Figure 4A–C, respectively). When the stimulus was present, the iCHIKV all mAb doses inhibited the mechanical hyperalgesia at some time points. The dose of 5 µg of mAb1 and mAb2 presented higher inhibition of iCHIKV-induced mechanical hyperalgesia, and the doses of 1 and 5 µg of mAb3 presented similar efficacy of analgesia (Figure 4D–F). Thus, these results demonstrate the participation of E2 protein in pain caused by iCHIKV. Mock was inactive as compared to the saline group.

### 3.5. The Mechanical Hyperalgesia Caused by iCHIKV and rE2 Protein Was Inhibited by the Absence or Blockade of TRPV1 Receptor

**Figure 5:**
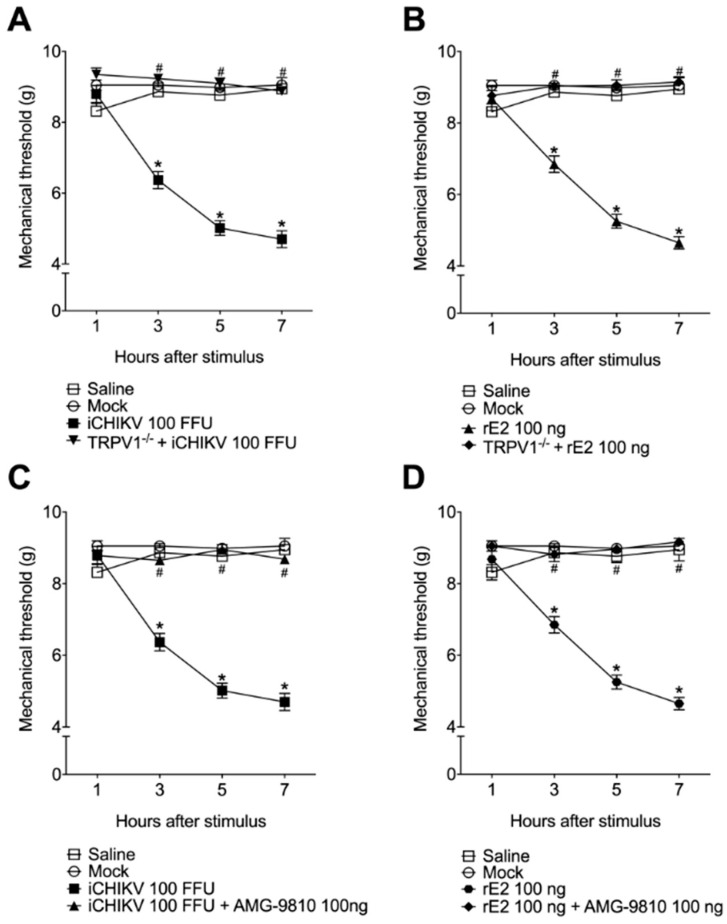
Targeting TRPV1 channels reduces iCHIKV and rE2 protein-induced mechanical hyperalgesia. iCHIKV (100 FFU, 5 µL, **A**), rE2 (100 ng, 5 µL, **B**), or Mock were injected (intra-articular) in C57-BL/6 and TRPV1−/− mice or iCHIKV (100 FFU, 5 µL, **C**), rE2 (100 ng, 5 µL, **D**) or Mock were injected (intra-articular) in C57-BL/6, and mice were treated with TRPV1 antagonist AMG-9810 (100 ng, intrathecal). The mechanical hyperalgesia was evaluated at 7 h after stimulus injection. Results are presented as mean ± SEM of six mice per group per experiment and are representative of two independent experiments. * *p* < 0.05 compared to Mock group; # *p* < 0.05 compared to stimulus group. ANOVA followed by Tukey’s test.

The TRPV1 knockout animals did not present mechanical hyperalgesia in response to stimulation with iCHIKV or rE2 (Figure 5A,B). We also observed that treating C57BL/6 mice with TRPV1 antagonist AMG-9810 (100 nmol, i.t.) 5 min after the stimulation with iCHIKV or rE2 prevented the increase in mechanical hyperalgesia (Figure 5C,D), suggesting the importance of this receptor in Chikungunya virus induced-pain.

### 3.6. iCHIKV and rE2 Protein Induce Thermal Hyperalgesia in Mice: Inhibition by Anti-rE2 Monoclonal Antibodies and by Targeting TRPV1 Receptor

**Figure 6:**
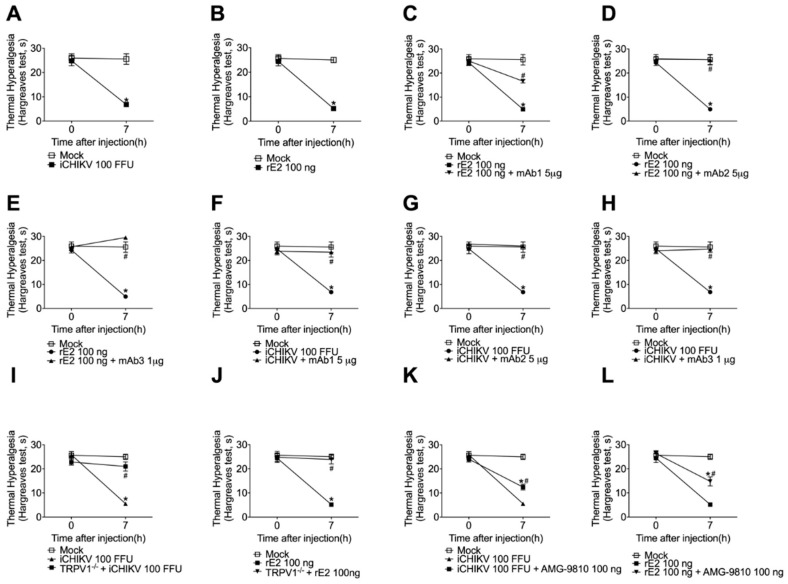
Anti-E2 monoclonal antibodies and targeting TRPV1 receptor inhibit iCHIKV- and rE2 protein-induced thermal hyperalgesia. iCHIKV (**A**) and rE2 (**B**) induce thermal hyperalgesia. mAb1 (5 µg, 5 µL, **C**,**F**), mAb2 (5 µg, 5 µL, **D**,**G**), and mAb3 (1 µg, 5 µL, **E**,**H**) were co-injected (intra-articular) with rE2 (100 ng, 5 µL, **C**–**E**), or iCHIKV (100 FFU, 5 µL, **F**–**H**) or Mock (control, 10 µL). iCHIKV (100 FFU, 5 µL, **I**), rE2 (100 ng, 5 µL, **J**), or Mock were injected (intra-articular) in C57-BL/6 and TRPV1−/− mice or iCHIKV (100 FFU, 5 µL, **K**), rE2 (100 ng, 5 µL, **L**) or Mock were injected (intra-articular) in C57-BL/6, and mice were treated with TRPV1 antagonist AMG-9810 (100 ng, intrathecal). The thermal hyperalgesia was evaluated at 7 h after stimulus injection. Results are presented as mean ± SEM of six mice per group per experiment and are representative of two independent experiments. * *p* < 0.05 compared to Mock group; # *p* < 0.05 compared to stimulus group. ANOVA followed by Tukey’s test.

After observing the participation of TRPV1 receptors in mechanical hyperalgesia induction, we aimed to evaluate the effect of the E2 protein in inducing thermal hyperalgesia since TRPV1 is involved in thermal hyperalgesia. We found that iCHIKV and rE2 protein induce thermal hyperalgesia showing a significant response after 7 h of stimuli (Figure 6A,B). Therefore, we treated animals with anti-rE2 monoclonal antibodies and observed a reduction in the thermal sensitivity after co-injection with rE2 protein (Figure 6C–E) or inactivated CHIKV (Figure 6F–H). The TRPV1 knockout animals were also tested for thermal hyperalgesia, and no significant increase in response after stimuli with iCHIKV or rE2 was observed (Figure 6I,J). We also observed that treating C57BL/6 mice with TRPV1 antagonist AMG-9810 (100 nmol, i.t.) 5 min after stimuli with iCHIKV or rE2 prevented the increase in thermal hyperalgesia (Figure 6K,L).

### 3.7. iCHIKV and rE2 Protein Activate TRPV1+ DRG Nociceptive Neurons

**Figure 7:**
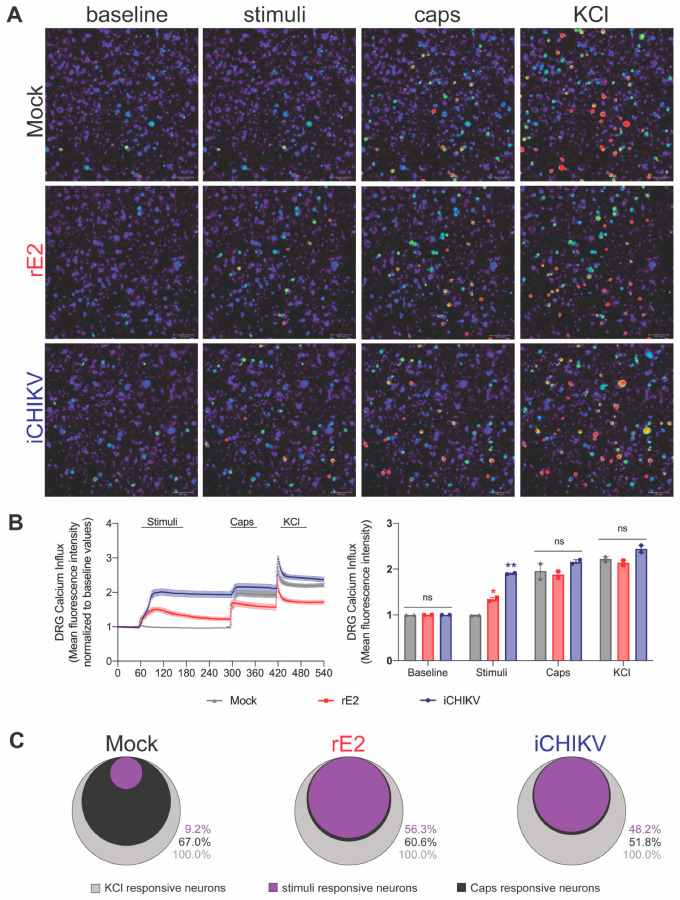
iCHIKV and rE2 activate DRG neurons, demonstrating the direct neuronal effect. Naïve DRG neurons were collected and processed for neuronal isolation and culture. DRG neurons were loaded with Fluo-4AM as a probe sensor for intracellular calcium levels representing neuronal activation. DRG neurons were recorded at basal conditions upon stimulus with iCHIKV (100 FFU, 10 µL), rE2 (100 ng, 10 µL), or Mock (control, 10 µL) and then stimulated with capsaicin (1 µM per dish), and afterward KCl control of depolarization. Representative images of DRG neurons upon each condition (**A**). Representative traces and bar representation of data (**B**). Venn diagram evidencing that iCHIKV and rE2 activated a population of TRPV1 responsive neurons (**C**). Image resolution: 288 × 220 mm (96 × 96 DPI). Results are expressed as mean ± SEM, *n* = 4 DRG plates (each plate is a neuronal culture pooled from six mice) per group per experiment, two independent experiments (* *p* < 0.05 vs. saline and mock; ** *p* < 0.05 vs. saline, mock and rE2; ns, not significant. one-way ANOVA followed by Tukey’s post-test).

The pharmacological and behavioral data of Figure 5 and Figure 6 demonstrate that TRPV1 channels are involved in iCHIKV and rE2-triggered mechanical and thermal hyperalgesia. To further support these results, we assessed whether iCHIKV and rE2 activate TRPV1+ neurons directly in DRG neuronal culture. This approach is important since it bypasses the contribution of non-neuronal cells in inducing hyperalgesia and directly demonstrates whether iCHIKV and rE2 can activate neurons. Figure 7A presents representative images of neuronal cultures upon iCHIKV and rE2 stimulation, followed by capsaicin stimulation and control of depolarization using KCl. Figure 7B presents the representative traces of calcium levels ± s.e.m. as well as the visualization of these results in bar panels (mean ± s.e.m.), which make it easier to observe that iCHIKV and rE2 activate DRG neurons. iCHIKV can even reach an activation level similar to capsaicin. Figure 7C shows that the neuronal population that responded to iCHIKV and rE2 was almost entirely responsive to capsaicin, thus demonstrating that iCHIKV and rE2 activate TRPV1+ neurons. Mock control elicited a small proportion of neuronal activation (Figure 7). These results of Figure 7 line up very well with the pharmacological and behavioral data of Figure 5 and Figure 6. Furthermore, Figure 7 data supports that the in vivo neuronal activation seen in Figure 3 involves, at least in part, direct neuronal activation by iCHIKV and rE2, although the exact receptor activated by them remains to be determined.

## 4. Discussion

Chikungunya fever is a disease well known for causing severe joint pain in almost the totality of infected patients, making pain a characteristic symptom of this infection [3]. Discomfort in performing the activities of everyday life (e.g., walking, eating, and getting dressed) can be present in about 50% of patients [9], so erratic, relapsing, and incapacitating long-lasting arthralgia is considered the hallmark of Chikungunya fever [13]. This scenario underlies the economic impact of CHIKV infection, a disease which, considering the globalization of its competent vectors, is at risk of becoming a major public health threat.

The E2 envelope protein has been appointed as one of the viral epitopes responsible for eliciting an immune response, causing joint inflammation and swelling [14,25]. However, it is not known whether the pain is associated with the E2 protein. Herein, we showed that the E2 protein is involved in Chikungunya virus-induced joint pain. We found that the injection of inactivated CHIKV induces mechanical and thermal hyperalgesia, and a similar effect was observed after stimulus with recombinant E2 protein. To our knowledge, this is the first demonstration that this protein induces pain.

We next interrogated neuronal activation by performing calcium imaging with DRG neurons dissected from mice infected with iCHIKV and rE2. An increase in calcium influx indicates DRG neuron activation [34]. Therefore, we demonstrated that iCHIKV and rE2 protein produce peripheral neuronal sensitization as observed by higher baseline levels of calcium influx in DRG neurons compared to Mock mice. Thus, peripheral neuronal sensitization might be responsible for the persistent pain observed herein. Other viruses, such as ZIKV and its envelope protein, are capable of inducing a slow increase in intracellular Ca^2+^ concentration mediated by TRPV4, and targeting this receptor results in reduced infectivity by dengue, hepatitis C and Zika virus [36]. In addition, a calmodulin inhibitor significantly reduced CHIKV replication in pretreated mice, indicating that calcium metabolism may have an important role in the kinetics of infection [37].

Previous studies have demonstrated that persistent and debilitating arthralgia is a frequent concern among patients with CHIKV infection, especially among middle-aged and elderly patients, the most affected group during epidemics [9]. At the acute stage of CHIKV fever, loss of productivity is a frequent concern, and more than 70% of affected patients usually take time off from work [18]. In the same way, chronic disease is characterized by severe and persistent arthralgia, which can last months or even years affecting the patient’s economic and social life [5,6,9].

The social and economic impact of CHIKV epidemics underlines the need for accurate diagnostics and effective antiviral and analgesic therapeutics since vaccines are not yet available [38]. Monoclonal antibodies have been used as standards for laboratory-based diagnosis of arthritogenic alphavirus diseases, including CHIKV, and have been developed in several countries [39]. The alphavirus E2 protein is thought to be involved in virus attachment to host cell receptors and contains critical binding sites for neutralizing antibodies [40]. In fact, a recent study described the generation and characterization of five monoclonal antibodies specific for the E2 glycoprotein of CHIKV without evidence of cross-reactivity with other alphaviruses, two of which provided complete protection against arthritis in a CHIKV mouse model when administered prior to infection [38]. Evidence also demonstrates that post-treatment with an anti-E2 mAb can reduce CHIKV viremia, edema, and lethality [19]. In this sense, we used treatment with three different types of anti-rE2 mAbs to demonstrate the control of rE2- and iCHIKV-induced pain. Thus, specific anti-E2 mAbs represent promising therapeutic tools for CHIKV disease, controlling both the virus binding to target cells (virus neutralization) [40] and pain triggered by the CHIKV-E2 antigen (present data). We performed a co-treatment protocol (anti-E2 mAbs plus iCHIKV or rE2) because a single bolus injection of the stimulus was used, and no active production of E2 protein occurred in these models (iCHIKV or rE2 injection). Thus, a post-treatment could result in losing the activity window of the anti-E2 mAbs since they act by inhibiting E2 protein binding to cells [40]. To our knowledge, this is the first demonstration of the use of anti-E2 monoclonal antibodies in the treatment of pain in CHIKV disease, and that CHIKV-E2 antigen has a role in pain induction. These results also raise the possibility of using anti-E2 antibodies developed by other groups to reduce Chikungunya infection pain.

It is well known that the ion channel transient receptor potential vanilloid 1 (TRPV1) plays an important role in acute and chronic pain processing with a major role in thermal hyperalgesia, but also contributing to mechanical hyperalgesia, for instance, by TRPV1+ neurons’ release of neuropeptides [41,42,43,44]. Expressed predominantly by primary nociceptive neurons, the TRPV1 receptor is a ligand-gated, non-selective cation channel [45,46] associated with a rise in intracellular calcium [47]. The lack of data regarding mechanistic details involved in CHIKV-induced joint pain underscores a need to search for specific therapeutic targets to control it. Herein, as we observed iCHIKV and rE2 induce thermal hyperalgesia, we decided to investigate the participation of TRPV1 in Chikungunya pain. To test that, mice lacking TRPV1 received iCHIKV and rE2. We observed that both iCHIKV and rE2 did not induce mechanical or thermal hyperalgesia effects in TRPV1−/− mice. Similarly, AMG-9810 (TRPV1 antagonist)-treated mice did not present mechanical or thermal hyperalgesia in response to stimulation with iCHIKV and rE2. Strategies targeting TRPV1 are effective at reducing acute and chronic pain in different models [41,48,49], and TRPV1 antagonist AMG-9810 and others have been shown to efficiently attenuate thermal hyperalgesia induced under inflammatory conditions and increase the noxious heat threshold [50]. Lining up with these pharmacological, genetic ablation, and behavioral approaches, iCHIKV and rE2 activated in culture naïve TRPV1+ DRG neurons, demonstrating that both (iCHIKV and rE2) can directly activate nociceptor sensory neurons to cause pain. The receptor(s) used by iCHIKV and rE2 to activate TRPV1+ neurons remains to be determined. Of note, it has recently been shown that TNFα mediates pain, tissue damage, and viral clearance in CHIKV infection [51]. This study [51] focused on one cytokine that is produced by immune cells and can trigger nociceptor sensitization and pain [52]. Our study brings a different approach since it identified that the E2 protein could activate TRPV1+ neurons to cause pain and mediate CHIKV nociceptive activity.

The current mouse models do not entirely recapitulate the chronic pain caused by CHIKV, or this disease symptom has not been investigated. The field can evolve, for instance, by improving the models in terms of pain chronicity, using humanized mouse models [53], and applying human cells to translate the results further [54,55]. Virtually every disease model has its qualities and limitations; we recognized that we did not use viable CHIKV infection, and the current models we developed (iCHIKV or rE2 injection) do not present a chronic pain profile by a single injection. Despite these limitations, our experimental approach was adequate to unveil a novel disease mechanism, e.g., the role of the E2 protein from CHIKV in activating TRPV1+ neurons to induce pain. This was possible because we used iCHIKV and rE2. If we treated mice with anti-E2 antibodies in ongoing CHIKV infection, we could not distinguish the role of E2 protein as an antiviral or analgesic target since anti-E2 antibodies would reduce infection [19,38]. Further pre-clinical and clinical data can emerge based on the current findings towards the development of novel analgesics to treat chronic CHIKV pain. One potential experimental approach, to be brief, could be the use of human stem cell-derived TRPV1+ sensory neurons [54] paired or not with human stem cells-derived sensory neurons lacking functional TRPV1 channel [55] to bring a translational perspective by investigating in human cells the neuronal mechanisms demonstrated herein in mice.

## 5. Conclusions

**Figure 8:**
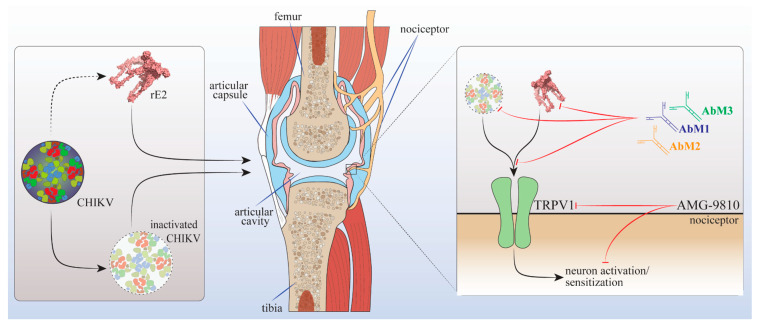
Schematic summary proposal of Chikungunya virus-induced pain. Intra-articular administration of inactivated Chikungunya virus (iCHIKV) and recombinant E2 (rE2) protein induce mechanical and thermal hyperalgesia in Swiss mice and C57BL/6 mice. Treatment with the three anti-E2 antibodies (mAb1, mAb2, and mAb3) reduced iCHIKV- and rE2-induced hyperalgesia demonstrating the contribution of E2 to the pain caused by the Chikungunya virus. iCHIKV and rE2 pain depend on direct activation of TRPV1+ DRG neurons as observed by genetic ablation (TRPV1 deficiency), pharmacological treatment (AMG-9810), behavioral analysis, and neuronal activity.

Taken together, our findings demonstrate that E2 protein is an important component in Chikungunya virus-induced hyperalgesia and targeting it can be a promising approach to treating joint pain after CHIKV infection, as demonstrated using anti-E2 mAbs. The mechanism of hyperalgesia induction by CHIKV and its E2 protein is, at least in part, dependent on the activation of TRPV1+ primary afferent neurons whose cellular bodies are in the DRG. Moreover, we demonstrated that targeting the TRPV1 receptor can be a promising therapeutic approach to managing CHIKV-induced pain, as summarized in Figure 8.

## Data Availability

Authors should be contacted for data and materials requests.
